# Ethical considerations for non‐procreative uterus transplantation

**DOI:** 10.1111/bioe.13379

**Published:** 2024-11-14

**Authors:** J. Y. Lee

**Affiliations:** ^1^ Department of Public Health University of Copenhagen København Denmark

**Keywords:** assisted reproductive technologies, organ transplantation, procreation, reproductive ethics, uterus transplantation, UTx

## Abstract

The growing demand for uterus transplantation (UTx) invites continued philosophical evaluation of the *function* of UTx (and what constitutes its ‘success’), as well as the recipient *eligibility* for UTx. Currently, UTx caters to partnered, cisgender women of childbearing age looking to get pregnant and give birth to a biogenetically related child. The medical justification for this—the treatment of uterine infertility—explains the primacy of this practice. However, this dominant conceptualization of UTx does not necessarily capture the diverse needs for which both cis‐ and transgender women might take interest in UTx that are not strictly procreative, such as bodily integrity and gender identity reasons. In this paper, I argue that non‐procreative motivations for uterus acquisition ought to be taken seriously as a matter of non‐discrimination and consistency.

## 1 INTRODUCTION

Uterus transplantation (UTx) is a surgery in which a uterus from a live or deceased donor is transferred to eligible women with absolute uterine factor infertility (AUFI), to enable pregnancy and childbirth. While UTx is in some ways comparable to standard solid organ transplantation, it is a nonessential and non‐life‐saving procedure^1^ that is unique in that “the aim of the graft is to function for a finite number of years until the woman has completed her family.”^2^ Thus, UTx is the world's first *ephemeral* transplant, as the uterus must be taken out of the body after completion of childbearing (practically speaking, 1–2 pregnancies) to relieve the recipient of the side effects of taking immunosuppressive medications.^3^

To date, it is said that some 100 surgeries (and around 50 live births) have taken place around the world.^4^ Most such surgeries—which may cost upwards of $300,000 in the US—have been funded through clinical trials. Despite ongoing reservations in the bioethical literature about the funding,^5^ medical,^6^ and relational^7^ implications of UTx, continued advancements in the field are likely to take this experimental surgery into mainstream clinical practice.^8^ Baylor University Medical Center, in the United States, already offers UTx outside of a clinical trial—with recipients being asked to bear the costs of the surgery themselves.^9^ As UTx becomes more widely available in such ways, however, opportunities to ethically reflect on prevailing practices arise—including questions about what the *objectives* of UTx should be and *who* should be able to access it. For this article, therefore, I am interested in critically examining these questions.

Without providing an *endorsement* of UTx wholesale, my goal is rather to explore how the ethics of UTx access could be improved in the context of its growing worldwide popularity, without recommending illiberal measures. My perspective should therefore be of interest to those who are for UTx, against UTx or somewhere in between. By uncovering the strictly *procreative* practices of UTx currently upheld by bioethicists and practitioners, I will show that other medically legitimate interests that people might take in acquiring a uterus—for reasons that go beyond gestation and childbirth—have been underappreciated in UTx ethics. As UTx becomes more common and made available outside of research settings, or feasible in individuals other than cisgender women with AUFI, it makes sense to challenge the procreative norms underlying the rationale for UTx and to begin thinking about accommodating hopeful recipients more inclusively. The contribution that I make on this topic will therefore be to argue that we have reasons of non‐discrimination and consistency to take the idea of UTx done for *non*‐procreative ends seriously.

## 2 WHAT—AND WHO—IS UTX FOR?

The standard justification of UTx as a practice is that it will “improve quality of life in women with AUFI who desire to carry their own pregnancies.”^10^ This medical need appears to be cashed out via “estimates of the numbers of women with uterine infertility that are likely to ‘be interested in conceiving’.”^11^ UTx has been described as “the only option to restore reproductive anatomy and functionality”^12^ in context. On this view, the function of UTx is to offer a *medical* treatment for infertility in cisgender women of childbearing age who have absolute uterine factor infertility (AUFI). AUFI may either be congenital (Mayer–Rokitansky–Küster–Hauser Syndrome) or acquired (hysterectomy).

Furthermore, since natural conception is not possible with a transplanted uterus, this surgery is always offered alongside assistive reproductive techniques like In Vitro Fertilization (IVF), by which an embryo will be created from (typically) the recipient's own egg and her partner's sperm.^13^ The embryo would then be implanted in the recipient after a stable uterus graft is established—generally at least 6 months or so after the surgery.^14^ Indeed, much of the recipient inclusion criteria for UTx revolve around its procreative viability in the individual, with childbearing age (ideally under 38) and “‘willingness to undergo IVF” being primary conditions for patient participation.^15^ In some cases like in the UK trial, a recipient must have created a minimum of ten embryos before undergoing transplantation.^16^ These conditions for recipient eligibility are unsurprising, given that UTx is treated as the only means by which women with certain kinds of uterine infertility might bear children of their own.

Bayefsky and Berkman have proposed a ranking system for uterus allocation that captures the general consensus that reproductive motives go hand in hand with restoring uterine anatomy to justify UTx; according to them, it is not enough for a woman with AUFI to want to “restore bodily integrity” through a uterus transplant. The recipient in question must, at the same time, be motivated to “experience pregnancy and become a mother.”^17^ It is on this basis that they have also suggested prioritizing UTx for women who are most likely to be able to provide well for their children, for instance by giving the latter a stable and safe environment to grow up in.^18^

Suffice to say, then, that the main objective of UTx currently—and its ultimate measure of success—is biogenetic pregnancy and childbirth for the recipient. Of course, this standard clearly builds in age‐, cis‐ and hetero‐normative principles: access to surgery is effectively limited to partnered, young, cisgender women with a certain type of uterine infertility who wish to gestate a child biogenetically related to them. Some of these norms may be changing, however: while recipients are for the most part expected to be in stable relationships, the potential inclusion of single women (who are standardly excluded) has been considered on the condition that they have a good support system, are “…willing to undergo an IVF cycle with oocyte freezing or embryo freezing with donor spermatozoa,” and “plan to achieve pregnancy within a year after their UTx procedure to avoid prolonged immunosuppressive treatment.”^19^ Thus, single women may be seen as next in line to be treated as appropriate recipients for UTx, if they are enthusiastic about the procreative potential of UTx.

This core goal and value of biogenetic pregnancy—the procreative objective of UTx—carries into current discussions about UTx *beyond* cisgender women with AUFI. Bioethicists have already begun speculating about whether “trans women and men can also assert a right to gestate, should science conquer this last frontier.”^20^ Whilst UTx in transgender persons has no medical precedent (yet), some have already claimed that the first uterus transplant in a transgender woman can be expected in the next few years, if not sooner.^21^ The literature on UTx increasingly supports the inclusion of transgender women, because they are viewed as suffering from a comparative lack of ability to conceive and gestate.^22^ After all, if UTx has been developed in order to “…ameliorate unhappiness caused by a discrepancy between procreative ability and reproductive aspirations,”^23^ there is no reason why those who are genetically XY, such as transgender women and women with complete androgen insensitivity syndrome, should not be considered potential UTx candidates as well.^24^ Others have also called for the potential inclusion of all non‐women—such as cisgender men, intersex people, nonbinary people and transgender men^25^—to decouple gender identity from gestation.

In general, I am in broad agreement with calls towards greater inclusivity—after all, it may not only be cisgender women with AUFI who are interested in UTx for procreative reasons. Whatever one's personal position may be on the value of biogenetic procreation, this kind of family‐making *is* considered important enough for major public health entities like the World Health Organization to claim that “everybody has the right to choose to whether to have children.”^26^ If what UTx endeavours to fulfil is the individual desire to gestate and bear children, or to at least alleviate the suffering that may arise in those who want but are unable to do so, it would seem that we have reasons of non‐discrimination to include *anyone* who is similarly affected by these issues (provided that the surgery is technically feasible and available). We can plausibly make such claims on the assumption that “medical science has no bearing in determining what an unreasonable demand is from a person of sound mind, except when it is about medical and surgical risk and the allocation of resources.”^27^ In other words, the kinds of experiences that are thought appropriate to be addressed within healthcare settings—such as infertility—are to some extent determined by people's “existential suffering”^28^ and the priority we assign to the alleviation of such suffering.

It is still possible, in principle, to push the boundaries of *what* and *who* UTx is for even further. While I take no issue with the fact that UTx is generally done for childbearing purposes, my view is that it should not necessarily be *limited* to such a purpose. This is because UTx is already a unique example of a surgery that finds its justification not in its medical necessity or life‐saving potential, but rather in its potential to improve *quality* of life.^29^ It is a novel way to expand the procreative choices and prospects of people who lack a uterus, which can be construed as an enhancement of reproductive autonomy.^30^ Bioethicists already accept that it may be inconsistent or discriminatory not to recognize or assist people in their desires for biogenetic parenthood, should they struggle to conceive.^31^ Based on research that has already been done on narrativizations of the uterus by ciswomen with AUFI and transwomen especially (which I elaborate in the next section, ‘Non‐standard values of the uterus’), it will emerge that UTx contains *non*‐procreative life‐enhancing potential as well, for instance, as a mode of treatment to attain alignment between bodily integrity and gender identity. This is not to say that what I am claiming as “non‐procreative” henceforth cannot *overlap* with “procreative” motives, or to say that one type should be prioritized over the other. Rather, I use the term “non‐procreative” simply to extend the purview of legitimate reasons that any person might have to desire a uterus. This is intended to designate the potential value of conducting the surgery for ends other than childbirth.

## 3 NON‐STANDARD VALUES OF THE UTERUS

For the remainder of this paper, I shall provide support for the novel bioethical argument that there are reasons of non‐discrimination and consistency to potentially consider *non‐*procreative UTx as a medically and morally justified surgery. It is not, currently, possible for otherwise eligible recipients to be cleared for UTx simply to acquire a uterus as an end in itself. As mentioned earlier, the standard process for a uterus graft assumes biogenetic motivations and the extraction of eggs/creation of embryos is a necessary condition of participating in clinical UTx trials as a recipient. However, I will illustrate that this practice unwarrantedly marginalizes other functions and values of the uterus, on the basis of which one might wish to acquire a uterus. My critical analysis in this section will thus enable me to establish the first horn of my argument: namely, that we have reasons of non‐discrimination to consider non‐procreative UTx as an option.

Undoubtedly, the uterus plays an essential role in enabling and regulating reproductive processes like conception and gestation. However, it is evident that acceptance of this standard description of the uterus can be asserted alongside (problematizable) social ideas or internalizations about what it *should* be used for: for example, that it is mandatory for those with a uterus to use it for childbearing,^32^ or that because the uterus can be used for gestation, it must therefore be functionally useless outside of that purpose. Indeed, these mistaken (and pronatalist) logics of idealization around the uterus as a procreative instrument have already manifested discriminatory consequences in the recruitment of live uterus donors. It has been argued elsewhere, for example, that child‐free women who may autonomously wish to donate their uterus are unjustly excluded from the opportunity to donate—because it is deemed inappropriate for women within “reproductive” age who have not “used” their uterus for childbearing to make decisions about giving it away.^33^ This parallels a well‐documented problem in reproductive healthcare: namely, the discrimination of autonomous women seeking sterilization who are denied such choices, especially relative to men, due to sexist and pronatalist norms about the necessity of childbirth for all capable women.^34^ In turn, this view can even reinforce the view that it should only be the mothers of potential recipients who ought to give away their uterus, since they would be older women done with childbearing and supposedly no longer in “need” of one.

Conflating the fact that the uterus *has* a reproductive function with the normative narrative that procreation must therefore be its sole value or end goal also has the effect of marginalizing other important values associated with the uterus, on the basis of which hopeful recipients might seek to acquire and keep a uterus. By highlighting some non‐standard perspectives about why the uterus may be valued below, I will illustrate how restricting UTx to procreative ends only would have discriminatory implications for those who have legitimate medical wishes to undertake UTx for different reasons.

First, let us acknowledge the weighty meanings of the uterus for women with AUFI, especially for women who were born without a uterus (e.g., those with Mayer–Rokitansky–Küster–Hauser Syndrome, or MRKH for short), relative to, say, women who were born with one but then had a hysterectomy later in life for medical reasons. MRKH is described as a “congenital anomaly of the female genital tract”^35^ featuring vaginal agenesis and uterine abnormalities (e.g., ‘absent’ or ‘underdeveloped’ uterus). This means women with MRKH struggle with penile–vaginal intercourse, do not menstruate and cannot carry a pregnancy. Being diagnosed with this condition can have a shocking and confusing impact, with “negative emotional reactions characterized by increased sensitivity to difference and impaired sense of femininity.”^36^ Feelings of alienation and being ‘different’ or ‘abnormal’ upon diagnosis can be exacerbated also by de‐personalizing encounters in the healthcare system and with healthcare professionals, who might use inappropriate language used to discuss the patient's so‐called abnormalities and carry out “distressing gynecological examinations.”^37^ Given this background, it is not surprising that persons with MRKH might view the possibility of receiving a uterus as a rare chance to restore feeling like a ‘normal’ woman again, as normative feminine identity is often anchored to “perceptions of normal physiology.”^38^ In other words, the very opportunity to acquire a uterus represents a meaningful prospect for agents with MRKH to restore a “normal” body in alignment with their gendered identity as ciswomen.

While the goal of restoring full fertility through UTx might certainly make up the majority of women with MRKH interested in the surgery, it should not be assumed that women in this group who fall outside of the eligibility margins, for example, due to having aged out of fertility treatment, would suddenly stop being interested in the prospect of receiving a uterus. Even if older women with MRKH cannot realistically hope to carry a pregnancy, having a uterus inside their body might still signify the hope to “achieve completeness as a woman.”^39^ Framing UTx as only justifiable when aimed toward the alleviation of suffering from infertility, then, unduly excludes other agents whose variant causes of suffering might also have been mitigated with UTx if only their needs were treated as comparably weighty.

Relatedly, there is the question of what happens to women with MRKH *after* they have successfully achieved childbirth with UTx. There is almost no information in the literature regarding the follow‐up of these women with respect to their desires to *keep* the uterus: it is simply asserted that a hysterectomy will be performed on them after completion of childbirth. I take this to mean that, whether the recipients approve or not, they will have no further opportunities to negotiate keeping a transplanted uterus inside the body after childbearing is complete. Yet, it may be that a woman who has already undergone childbirth via UTx might wish to avoid a transplant hysterectomy and prolong having the uterus inside her body, despite not necessarily wanting or being able to carry another child. This is because some women might experience “…loss and grief as a result of no longer having a uterus, coupled with a feeling of uncertainty regarding identity in terms of would they then return to view themselves again as a person with MRKH.”^40^

By granting women with MRKH the chance to receive one in the first place, the practice of performing mandatory hysterectomies on these recipients after childbirth may negatively disrupt conceptions of their very identity—as a person who must once again return to the state of not having a uterus. If transgender women were to be included in UTx, this may equally be an issue for them too, as they might anticipate “perceived potential worsening of dysphoric symptoms following hysterectomy.”^41^ Given the potential for grief and other subjectivities of loss to follow in patients who must have their transplanted uterus taken out, precluding any possibility to delay hysterectomy and to shift the end goals of UTx in accordance with the dynamic concerns of patients seems unfair—even to those who have already been afforded the privilege to successfully partake in procreative UTx.

The possibility of treating gender dysphoria also highlights transgender women's meaning‐making around the uterus. In one survey about transgender motivations for uterus transplants, it is established that transgender women would like to not only fulfil reproductive desires but also “concomitantly [alleviate]dysphoric symptoms, [enhance] feelings of femininity, and potentially [improve] happiness and quality of life”^42^ via UTx. It has been said that some transwomen may be interested in the potential for the uterus to fulfil a sense of “body completeness,”^43^ with the experience of *menstruation* representing a primary function and desideratum of the uterus. While I recognize that many agents who want to experience menstruation may wish to experience gestation as well, my point is that the multiple effects of the uterus may double up as gender‐affirming experiences that enhance transwomen's sense of femininity.^44^ For example, transwomen who have *already* completed childbearing prior to their transition may still have legitimate gender identity reasons (i.e., to relieve gender dysphoric symptoms and to affirm gender identity) to be interested in receiving a uterus. Perhaps a transwoman who is wary of the feasibility and safety of childbearing in genetically XY patients due to anatomical challenges^45^ would also be highly interested in having a uterus, just not for childbirth. Whatever the case may be, it would be wrong to simply presume that gestation is the only reason a transwoman could be legitimately interested in obtaining a uterus. Much like for ciswomen with MRKH, the possibility of UTx holds promise to medically help make one's bodily integrity congruent with one's gender identity. If UTx can plausibly help enhance gender congruence and alleviate dysphoric symptoms for transgender people in a way that is at least comparable to (if not more effective than) other available treatments like hormone therapy, UTx for bodily and identity‐affirming reasons should be recognized as medically significant.

Furthermore, even if UTx trials were open to transwomen desiring biogenetic procreation in principle, it is likely that legal barriers will for the near future continue to prevent them from achieving the aspect of childbirth, due to exclusion from the mandatory fertility treatment protocols. For example, patients may not be permitted to use embryos as transwomen^46^ or the option of cryopreservation might be unavailable to the transgender population generally.^47^ In view of these current challenges, it may be that receiving a uterus without fertility treatment is the more realistic scenario, assuming that the organ transplant itself is technically feasible. That is, given the life‐enhancing potential of the uterus as a gender‐affirming organ, transwomen may consider non‐procreative UTx to be the next best option, rather than to not receive a uterus at all. Broadening the scope of UTx to allow for non‐procreative practices under such specific circumstances could be a welcome option for transwomen who are already heavily discriminated in reproductive healthcare. Not allowing for the expansion of UTx scope at all, however, would further entrench their discrimination as agents with very restricted access to fertility treatment.

In sum, my position is that we ought at least in principle to consider the possibility of non‐procreative UTx as an alternative or additional option besides standard UTx, to outline a more inclusive and non‐discriminatory rendition of UTx. Non‐procreative UTx has the potential to medically address bodily and identity‐affirming concerns of many transwomen and ciswomen with MRKH, irrespective of whether they *also* wish for a child. Further, the non‐procreative option would constitute one way to counter the discrimination that transwomen already face in healthcare settings. While further empirical research would of course be needed to establish the more detailed health effects of performing UTx for non‐procreative reasons, the considerations that I have outlined in this section should lend credibility to the claim that doing this research would be medically significant in the first place.

## 4 IS NON‐PROCREATIVE UTX MEDICALLY JUSTIFIED?

As I established in the previous section, there are several groups for whom non‐procreative UTx is of legitimate interest due to its identity‐ and gender‐affirming potential, or even as an alternative to standard UTx in case there are external barriers to achieving pregnancy and childbirth. Even if one accepts the possibility that there could be legitimate medical interests for UTx outside of childbearing, however, one might still insist on not prioritizing or funding non‐procreative practices for UTx, perhaps on the assumption that procreative UTx has a better risk to benefit ratio as a type of infertility treatment. I will now cast doubt on this assumption by pointing out that it is the fertility‐driven aspects that *add* to the medical risks and costliness of the surgery. Moreover, I will show that non‐procreative UTx is not more or uniquely risky relative to standard UTx. In other words, it would be inconsistent to value procreative UTx over and above non‐procreative UTx, given certain facts about the actual comparative risks and costs. This will allow me to establish the second horn of my overall argument: namely, that there are consistency reasons to consider non‐procreative UTx as an option.

Let me contextualize my claims in view of the fact that a majority of the bioethical concerns surrounding UTx have to do with the burdensome effects of undergoing fertility treatment and undergoing pregnancy and childbirth under immunosuppressed conditions^48^—assuming that conception, pregnancy and childbirth are successful in the first place.^49^ Indeed, while anyone who has received a uterus must at all times take immunosuppressive drugs to minimize the prospect of organ rejection and so on, the regimen of immunosuppressive drugs often needs to be *increased* during pregnancy to maintain trough levels in a therapeutic window.^50^ This is on top of the fact that fertility treatments like egg extraction for IVF, to be done before the actual surgery, and going through implantation and pregnancy failure, can be highly burdensome physically and psychologically.^51^ Given these burdens, physicians are understandably divided over whether UTx in general is an acceptable medical intervention.^52^ All in all, it can take anywhere between 2 and 5 years to undergo UTx and have a successful birth,^53^ with the high‐risk pregnancy or pregnancies taking up much of this time‐consuming chronology. Thus, we should not be under the impression that acceptance of risk in procreative UTx must be *medically* more justified than UTx that does not involve the elements of fertility treatment or gestation.

Yet, this fertility‐centred practice of UTx *is* the norm currently permitted in certain research contexts and being trialled all around the world. For what it is worth, the risks of ethical concern are often accepted both by those wishing to receive the surgery (if we assume that they are rightfully exercising reproductive autonomy) and those responsible for carrying it out—if not the general public or medical community. Mats Brännström, who led the successful Swedish trials that culminated in the first successful live birth from UTx in 2014, has credited inspiration for his life's work to a hysterectomy patient of his, who, some 20 years ago, gave him the idea by saying: “I know the solution to the problem—you can transplant the womb from my mother.”^54^ Thus, we must also not underestimate the fact that patients are a driving force for its demand. Despite the risks associated with the surgery, existing research on the preferences of women with AUFI suggests that they would still strongly desire to try out UTx in spite of available alternatives like adoption and surrogacy.^55^ In contexts where successful trials have taken place, UTx surgeons have themselves suggested that eligibility standards like recipient age and parity be reconsidered to broaden representation of the patient population, for instance, to meet the needs of those who have had prior children or hysterectomies,^56^ who currently do not have priority for uterus allocation.

Funding, doing and participating in UTx research for the purposes of pregnancy and biogenetic procreation—which in itself receives “little questioning of the foundation of such a desire”^57^—*already* materialize a high degree of risk to not only recipients and donors but even the foetus.^58^ The negotiation of the ethical risk of procreative UTx has, evidently, been helped to a large extent by the strongly pronatalist and biogeneticist goals espoused by both the target patient group and the advocacy of some healthcare professionals working with ART and reproductive medicine. Yet, the potential asymmetry of risk acceptance here is especially concerning when it is far from clear that non‐procreative UTx would have a worse risk profile than procreative UTx. One obvious point in support of my claim to consider doing UTx for non‐procreative purposes would be that no radically new steps would be required. To the contrary, because non‐procreative UTx would minimize fertility treatment, it would be possible to cut down on the time, monetary and psycho‐physiological costs associated with processes like recipient egg extraction, the creation of embryos using IVF and, of course, the potential complications of pregnancy and C‐section. The task of navigating the risk of doing a uterus transplant for purposes beyond procreation would instead have to focus on the justification of the transplant itself and setting an appropriate timeline for keeping the uterus in the body under conditions of immunosuppression.

One might object here that the immunosuppressive burdens accepted for the purposes of procreative UTx have to do with the fact that the hopeful outcome—the birth of a healthy child—is the prospective *good* that will make the attendant medical vulnerabilities “worth” the risk and the cost of doing the surgery. The idea of carrying out UTx without the goal of childbirth, on the other hand, might be criticized due to a putatively less favourable risk‐to‐benefit ratio of immunosuppression. Yet, it is no secret that around half (or less) of UTx–IVF procedures actually result in a live birth,^59^ so plenty of the surgeries justified on the hope of childbirth will end up falling short of the goal anyway. Indeed, some of the unsuccessful cases of standard UTx have been due to repeated miscarriages and implantation failures over a number of years.^60^ In light of this, it would be reasonable to expect non‐procreative motivations for UTx to be considered and included, especially since non‐procreative UTx is simply more likely to be technically successful than procreative UTx by not eliciting the additional steps, expectations, attempts and time required to achieve childbirth.

Another valid doubt about non‐procreative UTx, however, may be that it does not establish a clear timeline for having a transplanted uterus. In contrast to procreative UTx, which provides a non‐arbitrary minimum time frame to go from transplant to childbirth to hysterectomy, some may worry that opening up opportunities to do UTx for non‐procreative reasons will introduce uncertainties regarding the threshold of time that should be seen as acceptable for any one person to have a transplanted uterus. As Jones et al. point out, “If uterus transplant is performed to allow women to experience menstruation and enhance perceptions of femininity, the duration of the graft would likely increase, significantly worsening its risk‐benefit profile.”^61^ I agree that this may pose a novel problem. However, the level of risk would really depend on whether the uterus is kept for a longer, or even indefinite, duration of time relative to procreative UTx. I am not necessarily claiming that we should allow for *permanent* uterus transplants, which would require life‐long immunosuppression and may well be weighed unfavourably.^62^

A time‐limited uterus transplant that would not require life‐long immunosuppression, or better still a transplant that would not be kept in the body any longer than a ‘standard’ uterus transplant, might be more ethically palatable. Establishing menstruation with UTx can take several months after the transplant anyway and happens to already be a precondition of moving on to the embryo transfer phase to attempt conception. Indeed, it is said that it could take anywhere between 6 and 12 months after surgery before embryo transfer is enacted for procreative UTx, “depending on the medical stability of the participant and the health of the uterus transplant.”^63^ This being the case, therefore, it does not seem totally out of the question to, as a minimum, simply allow for a stretch of time (say, up to the 5 years on average that recipients would have the uterus in their body for standard UTx) where the uterus is left in the recipient's body—of course at the choosing of the recipient and with their informed consent—for purposes outside of childbearing, like the alleviation of gender dysphoria. Of course, setting a standardized time limit on a transplanted uterus that is not being “used” for childbearing might seem arbitrary; a sensible way to approach this issue could be to weigh up the risks and benefits on a case‐by‐case basis and to also explore with the candidate recipient whether something like the mitigation of gender dysphoria can be done without actually having to resort to non‐procreative UTx. In any case, it should now be clear that permitting people to access non‐procreative UTx in a limited number of justified cases, despite some of the uncertainties that could be introduced as a result, would not necessarily have to provoke radically different outcomes or risks than what is already expected with standard UTx.

All in all, I have shown in this section that it may be a mistake to merely assume that there is a better warrant for procreative UTx over non‐procreative UTx‐from a therapeutic standpoint. On the contrary, many of the risks and costs that accompany UTx come from the procreative aspects of fertility treatment and childbirth. Keeping this in mind, we can plausibly conceptualize time‐limited versions of non‐procreative UTx that might be acceptable both because it minimizes the harms expected (and accepted) in standard UTx *and* because it may serve people who have medical conditions other than infertility in scenarios where an analogous need for treatment can be established (e.g. gender dysphoria cases). One who ethically supports UTx done for procreative reasons already should, for reasons of consistency, be committed to at least consider the possibility that a comparable version of non‐procreative UTx may be similarly justifiable.

## 5 PROBLEMS WITH NON‐PROCREATIVE UTX

While I have now outlined reasons that may justify expanding UTx objectives to include non‐procreative goals, I want to acknowledge that implementing non‐procreative UTx may of course be accompanied by ethical costs. In this section, therefore, I will reflect on anticipated objections and endeavour to defend my proposal from them. I venture to assuage sceptical readers by showing that the potential objections that might be levelled at my proposal are not insurmountable issues.

One pitfall of my account might be framed from a feminist critique of the norms enabled by reproductive technologies. In an increasingly pronatalist world, feminists are already worried that ART would not necessarily empower women of reproductive age with greater choices; rather, the fact that women have opportunities to use technologies to counter infertility (e.g., IVF), or even to preserve fertility (e.g., oocyte cryopreservation), might generate pressure on them to produce biogenetically related children one way or other.^64^ After all, bioessentialist norms about women take for granted that they ought to be prescribed primary childbearing and childrearing duties.^65^ Procreative UTx, then, would be another example of an ART that appears to reiterate the social mandate that women *should* get pregnant and have children at some point in their lives.^66^ Non‐procreative UTx, at least, might avoid this criticism, since it would not require recipients to fulfil the so‐called “motherhood mandate.”^67^ On the other hand, non‐procreative UTx could still be problematized for upholding a kind of gender identity mandate: after all, why nominate the uterus as the ultimate locus of feminine identity or treat it as a condition of womanhood?^68^ I had earlier claimed that granting gender‐affirming experiences such as menstruation should be recognized as a legitimate medical way to address something like gender incongruence or dysphoria; the downside of so doing, however, may be that I am implicitly importing a narrow ideal of femininity or coding certain embodied experiences as *essentially* or *necessarily* feminine.

I share these wider societal concerns about the role that enabling UTx might play in propping up oppressive and essentialist expectations for persons identifying as women to embody so‐called biologically female qualities. However, it is also important to recognize that we do not live in an ideal world, where it would be reasonable to expect women to be totally immune to influence from oppressive gendered norms. Furthermore, it is especially important not to let such considerations get in the way of providing gender‐affirming care, which is crucial for the health of transgender people.^69^ At the individual level, therefore, it seems sensible to remain tolerant and neutral with respect to personal intentions to pursue interventions like UTx, at least if our goal is not to issue illiberal measures as a response. The feminist critique, therefore, does not seem sufficient to *forbid* access to UTx in general (or indeed any other ART), assuming that it is available, feasible, sufficiently safe, strongly desired and likely to actually enhance the lives of those who seek it. This seems especially important when we consider that procreative UTx has already been normalized as a helpful tool for women, despite its inherent reification of pronatalist norms and roles. My point is not so much that I think it is intrinsically good or necessary in general for women to desire and seek childbirth, menstruation and so on, but rather that it would be hypocritical for anyone to treat this feminist critique as a decisive reason to block non‐procreative UTx while procreative UTx continues to be practiced.

Another major concern with my proposal of non‐procreative UTx might have to do with a potentially increased demand for live donors. While I have mainly focused on benefits to potential recipients that might follow from more inclusive practices of UTx, it must be acknowledged that no version of UTx could be said to be straightforwardly beneficial to live donors.^70^ Even on an optimistic view where we might hope that donors benefit psychologically from being able to help others or by contributing to science,^71^ the effects of undergoing a radical transplant hysterectomy are likely to impact live donors' quality of life.^72^ Of course, it may also be that the transplant is not successful, in which case the welfare prospects of live donors might be even worse. Except for a narrow class of cases in which people might already have independent reasons to dispense with their uterus that have nothing to do with helping others—for example, transitioning transgender men who were planning to undergo a hysterectomy anyway^73^—it is difficult to conceive how a potential live donor would benefit by providing their uterus. On top of this, uteri demand outstrips uteri supply, as is typically the case with transplantable organs in general.^74^ With more inclusive criteria for UTx recipients, it seems like this pre‐existing problem of organ scarcity would only be exacerbated. Given the standard view in medicine that patients generally have a negative right to *refuse* treatment but not necessarily a positive right (if there is one) to *demand* certain treatments,^75^ one would be hard‐pressed to justify placing any positive demand on ART third parties like live uterus donors to participate.

Thus, one apparent advantage of the more restrictive current practice of UTx for procreation would be that it simply requires fewer live donors and does not normalize a need to mobilize uterus donors as a potential solution to people's diverse welfare concerns. Be that as it may, it is clear that the problem of demandingness on live donors is not unique to my proposal for non‐procreative UTx. Harms to live donors are, in my view, one of the strongest objections to UTx in general, and the end goal of procreation does not exempt standard UTx from the same criticism. Indeed, the issue of harms to third parties in ART has been a huge cause for ongoing ethical debate also in the case of surrogacy, to varying degrees.^76^ In view of such concerns, I am of the view that no unwilling or uninformed third party should be exploited, manipulated or compelled into dispensing with their bodily materials and labour for ART practice—no matter what the end goal may be. It is of course difficult to guarantee such principles in the field of reproductive medicine, which tends to primarily use women's bodies to serve the needs of those with an interest in ART. However, the possibility for third parties to be harmed or treated unjustly should serve as a springboard for ideas about how to best diminish such harms or wrongs, rather than as a reason to forbid certain versions of UTx relative to others. To this end, some have even considered, for instance, fairer compensation of uterus donors as a way to avoid perpetuating harms on them.^77^ Another way to minimize pressure or emotional coercion of live donors could be to allow for enthusiastic and well‐informed *non*‐directed donors to be allowed to participate,^78^ which may be an ethically acceptable way to alleviate issues of uteri shortage, compared with requiring recipients to have to approach their own family members for a uterus. Whether UTx is done for procreative purposes or otherwise, measures that can mitigate harms or wrongs to third parties should be implemented in any case, to improve baseline ethical standards of doing UTx. So long as there are legitimate health and welfare considerations that motivate pursuit of UTx, and willing recipients and donors who understand and accept its implications, it does not seem such a stretch to simply extend this tolerance for those who are interested in UTx beyond procreative goals in places where this surgery is legal and safely practiced.

## 6 CONCLUSION

My aim in this article was not to dispute what has been put forward as the chief motivation of UTx in the first place: the pregnancy and birth of a healthy child. However, I wanted to reconsider the ethical assumptions around why there is currently no precedent for UTx *without* procreative intent. By proposing the idea of non‐procreative UTx, I opened up a new line of debate regarding the legitimacy of prevailing UTx practices and their ethics. I have argued that there are grounds of non‐discrimination and consistency to at least consider, if not permit, non‐procreative UTx—in *addition* to procreative UTx—in order to cater to a wider range of people who may have diverse interests in acquiring a uterus. To this point, I considered the potential benefits of offering non‐procreative UTx as a treatment option to a range of normally excluded candidates interested in having and keeping a uterus. I then showed that the potential risks and costs of carrying out UTx for non‐procreative reasons are not necessarily unique or worse relative to the risks that are *already* required to carry out procreative UTx. Finally, I acknowledged that while novel uncertainties associated with non‐procreative UTx could raise valid scepticism and cautionary measures, they did not constitute reasons to exclude my proposal from serious consideration.

## Footnotes

^1^
Jones, B. P., Kasaven, L., Vali, S., Saso, S., Jalmbrant, M., Bracewell‐Milnes, T., Thum, M‐Y., Quiroga, I., Friend, P., Diaz‐Garcia, C., Ghaem‐Maghami, S., Yazbek, J., Lees, C., Testa, G., Johannesson, L., Jones, B., & Smith, J. R. (2021). Uterine transplantation: Review of livebirths and reproductive implications. *Transplantation, 105*(8), 1695–1707. https://doi.org/10.1097/TP.0000000000003578

^2^
Jones, B. P., Kasaven, L. S., Chan, M., Vali, S., Saso, S., Bracewell‐Milnes, T., Thum, M. Y., Nicopoullos, J., Diaz‐Garcia, C., Quiroga, I., Yazbek, J., & Smith, J. R. (2022). Uterine transplantation in 2021: Recent developments and the future. *Clinical Obstetrics and Gynecology*, *65*(1), 4–14. https://doi.org/10.1097/GRF.0000000000000680

^3^
Benedet, S. (2019). Uterus transplantation fact sheet. *Acta Obstetricia et Gynecologica Scandinavica*, *98*, 1205–1206. https://doi.org/10.1111/aogs.13674; Dahm‐Kähler, P., Diaz‐Garcia, C., & Brännström, M. (2016). Human uterus transplantation in focus. *British Medical Bulletin*, *117*, 69–78. https://doi.org/10.1093/bmb/ldw002

^4^
Texas Children's Hospital. (2024). *Uterus Transplant Research Program.* https://www.texaschildrens.org/departments/uterus-transplant-research-program; Forbes, R. C., & Karp S. (2022). Uterine transplant—Progress, but concerns remain. *JAMA Surgery*, *157*(9), 797–798. https://doi.org/10.1001/jamasurg.2022.2652

^5^
Lotz, M. (2021). Public funding of uterus transplantation: Deepening the socio‐moral critique. *Bioethics*, *35*, 664–671. https://doi.org/10.1111/bioe.12914

^6^
Veroux, M., Giambra, M., Roscitano G., Giaquinta, A., Settaci, F., & Veroux, P. (2024). A new frontier in living donor transplantation: Uterus transplantation. *European Journal of Transplantation*, *2*(1), 57–65.

^7^
Lee, J. Y. (2023). Who should provide the uterus? The ethics of live donor recruitment for uterus transplantation. *Journal of Medical Ethics*. https://doi.org/10.1136/jme-2023-109227

^8^
Brännström, M., Racowsky, C., Carbonnel, M., Wu, J., Gargiulo, A., Adashi, E. Y., & Ayoubi, J. M. (2023). Uterus transplantation: From research, through human trials and into the future. *Human Reproduction Update*, *29*(5), 521–544. https://doi.org/10.1093/humupd/dmad012

^9^
Baylor Scott & White Health. (2024). Uterus transplant. https://www.bswhealth.com/treatments-and-procedures/uterus-transplant

^10^
Wall, A. E., Testa, G., Axelrod, D., & Johannesson, L. (2021). Uterus transplantation‐questions and answers about the procedure that is expanding the field of solid organ transplantation. *Baylor University Medical Center Proceedings*, *34*(5), 581–585. https://doi.org/10.1080/08998280.2021.1925064

^11^
Catsanos, R., Rogers, W., & Lotz, M. (2020). The ethics of uterus transplantation. In A. L. Caplan & B. Parent. (Eds.), *The ethical challenges of emerging medical technologies* (pp. 449–457). Routledge.

^12^
Etrusco, A., Fabio, M., Cucinella, G., Rossi, C., Billone, V., & Gullo G. (2022). The reproductive potential of uterus transplantation: Future prospects. *Acta Bio Medica: Atenei Parmensis*, *93*(2), e2022138. https://doi.org/10.23750/abm.v93i2.12868

^13^
In some cases, like in the United Kingdom, use of donor sperm is permitted.

^14^
Hopkins Medicine. https://www.hopkinsmedicine.org/gynecology-obstetrics/specialty-areas/uterine-transplant#faq

^15^
Ayoubi, J. M., Carbonnel, M., Racowsky, C., de Ziegler, D., Gargiulo, A., Kvarnström, N., Dahm‐Kähler, P., & Brännström, M. (2022). Evolving clinical challenges in uterus transplantation. *Reproductive BioMedicine Online*, *45*(5), 947–960.

^16^
Hammond‐Browning, N. (2019). UK criteria for uterus transplantation: A review. *BJOG BJOG*, *126*, 1320–1326.

^17^
Bayefsky, M., & Berkman, B. (2016). The ethics of allocating uterine transplants. *Cambridge Quarterly of Healthcare Ethics*, *25*, 350–365.

^18^
Ibid.

^19^
Ayoubi, J. M., et al., op. cit. note 15.

^20^
Alghrani, A. (2016). Uterus transplantation: Does procreative liberty encompass a right to gestate? *Journal of Law and the Biosciences*, *3*(3), 636–641. https://doi.org/10.1093/jlb/lsw048

^21^
Richards, E. G., Ferrando, C. A., Farrel, R. M., & Flyckt, R. L. (2023). A “first” on the horizon: The expansion of uterus transplantation to transgender women. *Fertility and Sterility, 119*(3), 390–391.

^22^
Balayla, J., Pounds, P., Lasry, A., Volodarsky‐Perel, A., & Gil, Y. (2021). The Montreal criteria and uterine transplants in transgender women. *Bioethics, 35*, 326–330. https://doi.org/10.1111/bioe.12832

^23^
Jones, B. P., Rajamanoharan, A., Vali, S., Williams, N. J., Saso, S., Thum, M. Y., Ghaem‐Maghami, S., Quiroga, I., Diaz‐Garcia, C., Thomas, P., Wilkinson, S., Yazbek, J., & Smith, J. R. (2021). Perceptions and motivations for uterus transplant in transgender women. *JAMA Network Open*, *4*(1), e2034561. https://doi.org/10.1001/jamanetworkopen.2020.34561

^24^
Sampson, A., Kimberly, L. L., Goldman, K. N., Keefe, D. L., & Quinn, G. P. (2019). Uterus transplantation in women who are genetically XY. *Journal of Medical Ethics, 45*, 687–689.

^25^
Wagner, D. N. (2023). For women only? Reconsidering gender requirements for uterine transplantation recipients. *Canadian Journal of Bioethics/Revue canadienne de bioéthique*, *6*(2), 53–65. https://doi.org/10.7202/1101128ar

^26^
World Health Organization. (2022). *Rights on having children.* https://www.who.int/tools/your-life-your-health/know-your-rights/rights-across-life-phases---early-and-middle-adulthood/rights-on-having-children

^27^
Zaami, S., Di Luca, A., & Marinelli, E. (2019). Advancements in uterus transplant: New scenarios and future implications. *European Review for Medical and Pharmacological Sciences, 23*, 892–902.

^28^
Bozzaro, C., Weismann, M., Westermann, A. M., & Alkatout, I. (2023). Is suffering a sufficient legitimation for UTx? *Bioethics*, *37*, 350–358. https://doi.org/10.1111/bioe.13135

^29^
Ejzenberg, D., Mendes, L. R., Haddad, L. B., Baracat, E. C., D'Albuquerque, L. A., & Andraus, W. (2016). Uterine transplantation: A systematic review. *Clinics (Sao Paulo*), *71*(11), 679–683. https://doi.org/10.6061/clinics/2016(11)10

^30^
da Graca, B., Johannesson, L., Testa, G., & Wall, A. E. (2022). The ethical evolution of uterus transplantation from concept to clinical procedure. *Clinical Obstetrics and Gynecology*, *65*(1), 24–36. https://doi.org/10.1097/GRF.0000000000000687

^31^
Tonkens, R. (2018). Infertilitism: Unjustified discrimination of assisted reproduction patients. *Monash Bioethics Review*, *35*, 36–49. https://doi.org/10.1007/s40592-018-0078-x; Murphy, T. F. (2019). Biogenetic ties and parent‐child relationships: The misplaced critique. *Bioethics, 33*(9), 1029–1034. https://doi.org/10.1111/bioe.12621

^32^
Lisle, L. (2022). Recognizing our womanhood, redefining femininity. In *Childfree across the disciplines: Academic and activist perspectives on not choosing children* edited by D. Thornley, (pp. 171–184). Rutgers University Press. https://doi.org/10.36019/9781978823129-012

^33^
Lee, op. cit. note 7.

^34^
Mikkola, M. (2017). Sex in medicine: What stands in the way of credibility? *Topoi*, *36*, 479–488. https://doi.org/10.1007/s11245-015-9350-3

^35^
Bean, E. J., Mazur, T., & Robinson, A. D. (2009). Mayer‐Rokitansky‐Küster‐Hauser Syndrome: Sexuality, psychological effects, and quality of life. *Journal of Pediatric and Adolescent Gynecology*, *22*(6), 339–346.

^36^
Facchin, F., Francini, F., Ravani, S., Restelli, E., Gramegna, M. G., Vercellini, P., & Aimi, G. (2021). Psychological impact and health‐related quality‐of‐life outcomes of Mayer–Rokitansky–Küster–Hauser syndrome: A systematic review and narrative synthesis. *Journal of Health Psychology*, *26*(1), 26–39. https://doi.org/10.1177/1359105319901308

^37^
Lou, S., Jensen, A. H., Vogel, I., Trolle, B., & Herlin, M. K. (2024). Understanding the diagnostic odyssey of women with Mayer‐Rokitansky‐Küster‐Hauser (MRKH) Syndrome in Denmark: A qualitative interview study. *Journal of Pediatric and Adolescent Gynecology*, *37*(4), 412–418.

^38^
Gilfillan, R., & Carter, P. (2024). Issues of identity, perceptions and isolation: An interpretative phenomenological analysis of women's experience of Mayer‐Rokitansky‐Küster‐Hauser (MRKH) syndrome. *Journal of Health Psychology*, *29*(3), 200–212. https://doi.org/10.1177/13591053231199253

^39^
da Graca, B., Johannesson, L., Testa, G., & Wall, A. (2021). Uterus transplantation: Ethical considerations. *Current Opinion in Organ Transplantation*, *26*(6), 664–668. https://doi.org/10.1097/MOT.0000000000000932

^40^
Järvholm, S., & Warren, A. (2022). Uterus transplantation: Lessons learned from a psychological perspective. *Clinical Obstetrics and Gynecology, 65*(1), 52–58. https://doi.org/10.1097/GRF.0000000000000673

^41^
Jones, B. P., et al., op. cit. note 23.

^42^
Jones, B. P., et al., op. cit. note 23.

^43^
Balayla, J., et al., op. cit. note 22.

^44^
Jones, B. P., et al., op. cit. note 23.

^45^
Polk, H., Johannesson, L., Testa, G., & Wall, A. (2022). The future of uterus transplantation: Cost, regulations, and outcomes. *Clinical Obstetrics and Gynecology, 65*(1), 101–107. https://doi.org/10.1097/GRF.0000000000000685

^46^
Hammond‐Browning, N. (2019). Uterine transplantation in transgender women: Medical, legal and ethical considerations. *BJOG: An International Journal of Obstetrics & Gynaecology*, *126*, 157. https://doi.org/10.1111/1471-0528.15482

^47^
Cheng, P. J., Pastuszak, A. W., Myers, J. B., Goodwin, I. A., & Hotaling, J. M. (2019). Fertility concerns of the transgender patient. *Translational Andrology and Urology*, *8*(3), 209–218. https://doi.org/10.21037/tau.2019.05.09

^48^
Zhang, R., Deng, S‐L., Lian, Z‐X., & Yu, K. (2020). Immunosuppression in uterine transplantation. *Transplant Immunology*, *63*, 101335. https://doi.org/10.1016/j.trim.2020.101335

^49^
Chung, R. K., Richards, E. G., Farrell, R., & Flyckt, R. L. (2022). Ascending toward a 30,000 foot view of uterus transplant. *Fertility and Sterility*, *118*(3), 586–587.

^50^
Johannesson, L., & Järvholm, S. (2016). Uterus transplantation: Current progress and future prospects. *International Journal of Women's Health*. *8*, 43–51. https://doi.org/10.2147/IJWH.S75635

^51^
Brännström, M., et al., op. cit. note 8.

^52^
Bortoletto, P., Hariton, E., Farland, L. V., Goldman, R. H., & Gargiulo, A. R. (2018). Uterine transplantation: A survey of perceptions and attitudes of american reproductive endocrinologists and gynecologic surgeons. *Journal of Minimally Invasive Gynecology 25*(6), 974–979.

^53^
Malasevskaia, I., & Al‐Awadhi A. A. (2021). A new approach for treatment of woman with absolute uterine factor infertility: A traditional review of safety and efficacy outcomes in the first 65 recipients of uterus transplantation. *Cureus*, 13(1), e12772. https://doi.org/10.7759/cureus.12772

^54^
Brännström, M. (2015). The Swedish uterus transplantation project: The story behind the Swedish uterus transplantation project. *Acta obstetricia et gynecologica Scandinavica*, *94*, 675–679.

^55^
Wall, A. E., et al., note. 10.

^56^
Johannesson, L., Giuliano, T., Menas, M. B., da Graca, B., Jessica R. W., Cristiano, Q., Nawar, L., Koji H., Elliott G. R., & O'Neill, K. (2023). Awareness and interest in uterus transplantation over time: Analysis of those seeking surgical correction for uterine‐factor infertility in the US. *Journal of Clinical Medicine*, *12*(13), 4201.

^57^
Catsanos, R., et al., op. cit. 11.

^58^
Ongun, H., Celik, K., Arayici, S., Dogan, N. U., Mendilcioglu, I., Ozkan, O., & Ozkan, O. (2024). Miracles of science: Birth after uterus transplantation. *Journal of Obstetrics and Gynaecology Research*, *50*(1), 5–14. https://doi.org/10.1111/jog.15825

^59^
Daolio, J., Palomba, S., Paganelli, S., Falbo, A., & Aguzzoli, L. (2020). Uterine transplantation and IVF for congenital or acquired uterine factor infertility: A systematic review of safety and efficacy outcomes in the first 52 recipients. *PLoS One*. *15*(4), e0232323. https://doi.org/10.1371/journal.pone.0232323

^60^
Brännström, M., Dahm‐Kähler, P., Kvarnström, N., Enskog, A., Olofsson, J. I., Olausson, M., Mölne, J., Akouri, R., Järvholm, S., Nilsson, L., Stigson, L., Hagberg, H., & Bokström, H. (2022). Reproductive, obstetric, and long‐term health outcome after uterus transplantation: Results of the first clinical trial. *Fertility and Sterility, 118*(3), 576–585.

^61^
Jones, B. P., et al., op. cit. note 23.

^62^
Ibid.

^63^
Penn Medicine. (2024). Penn fertility care. https://www.pennmedicine.org/for-patients-and-visitors/find-a-program-or-service/penn-fertility-care/

^64^
Donchin, A. (2015). The pronatalist imperative: Medicine, money, and markets. In *Procreation, power, and personal autonomy: Feminist reflections, unfinished manuscript* (pp. 1985–2014). https://hdl.handle.net/1805/7329

^65^
Purdy, L. M. (2019). Pronatalism is violence against women: The role of genetics. In W. Teays (Eds), *Analyzing Violence Against Women. Library of Public Policy and Public Administration* (Vol. 12). Springer. https://doi.org/10.1007/978-3-030-05989-7_9

^66^
O'Donovan, L., Williams, N. J., & Wilkinson, S. (2019). Ethical and policy issues raised by uterus transplants. *British Medical Bulletin*, *131*(1), 19–28. https://doi.org/10.1093/bmb/ldz022

^67^
Russo, N. F. (1976). The motherhood mandate. *Journal of Social Issues*, *32*, 143–153. https://doi.org/10.1111/j.1540-4560.1976.tb02603.x

^68^
Joshi, S. S. (2022). Metaphors of womanhood emerging in hysterectomy narratives. In *Handbook of aging, health and public policy: Perspectives from Asia* (pp. 1–11). Springer Nature Singapore.

^69^
Bhatt, N., Cannella, J., & Gentile, J. P. (2022). Gender‐affirming care for transgender patients. *Innovations in Clinical Neuroscience*, *19*(4–6), 23–32.

^70^
It should also be noted that the majority of UTx procedures use live donors, rather than deceased donors, due to the difficulties of procuring the latter.

^71^
Warren, A. M., McMinn, K., Testa, G., Wall, A., Saracino, G., & Johannesson, L. (2021). Motivations and psychological characteristics of nondirected uterus donors from the Dallas UtErus transplant study. *Progress in Transplantation*, *31*(4), 385–391. https://doi.org/10.1177/15269248211046027

^72^
Bruno, B., & Arora, K. S. (2020). Ethical implications of donor type for uterus transplantation: Why we should remain wary of using living donors. *The Yale Journal of Biology and Medicine*, *93*(4), 587–592.

^73^
Carbonnel, M., Léa K., Ninon C., Sophie L., Lucile P., Bernard C., Catherine, R., & Jean‐Marc, A. (2022). Transgender males as potential donors for uterus transplantation: A survey. *Journal of Clinical Medicine*, *11*(20), 6081. https://doi.org/10.3390/jcm11206081

^74^
Veroux, M., et al., op. cit. note 6.

^75^
Drane, J. F., & Coulehan, J. L. (1993). The concept of futility. Patients do not have a right to demand medically useless treatment. Counterpoint. *Health Prog*ress, *74*(10), 28–32.

^76^
Blazier J., & Janssens R. (2020). Regulating the international surrogacy market: The ethics of commercial surrogacy in the Netherlands and India. *Medicine, Health Care and Philosophy*, *23*(4), 621–630. https://doi.org/10.1007/s11019-020-09976-x

^77^
Lee, J.‐Y. (2023a). Ethics of live uterus donor compensation. *Bioethics, 37*, 591–599. https://doi.org/10.1111/bioe.13169

^78^
Jones, B. P., Rajamanoharan, A., Williams, N. J., Vali, S., Saso, S., Mantrali, I., Jalmbrant, M., Thum, M‐Y., Diaz‐Garcia, C., Ghaem‐Maghami, S., Wilkinson, S., Quiroga, I., Friend, P., Yazbek, J., & Smith, J. R. (2021). Uterine transplantation using living donation: A cross‐sectional study assessing perceptions, acceptability, and suitability. *Transplantation Direct*, *7*(3), e673. https://doi.org/10.1097/TXD.0000000000001124

